# Comparative study on three different methods for arm-span measurement: the Japan environment and Children’s study pilot

**DOI:** 10.1186/s12199-017-0632-9

**Published:** 2017-04-04

**Authors:** Mayumi Tsuji, Tadayuki Ayabe, Rie Tanaka, Ayako Senju, Eiji Shibata, Shunsuke Araki, Seiichi Morokuma, Masafumi Sanefuji, Koichi Kusuhara, Toshihiro Kawamoto

**Affiliations:** 1 Department of Environmental Health, School of Medicine, University of Occupational and Environmental Health, 1-1 Iseigaoka, Yahatanishi-ku, Kitakyushu, Fukuoka 807-8555 Japan; 2 Medical Support Center for Japan Environment and Children’s Study (JECS), National Center for Child Health and Development, Tokyo, Japan; 3 Regional Center for Japan Environment and Children’s Study (JECS), University of Occupational and Environmental Health, Kitakyushu, Japan; 4 Department of Obstetrics and Gynecology, School of Medicine, University of Occupational and Environmental Health, Kitakyushu, Japan; 5 Department of Pediatrics, School of Medicine, University of Occupational and Environmental Health, Kitakyushu, Japan; 6 Regional Centre for Japan Environment & Children’s Pilot Study, Kyushu University, Fukuoka, Japan

**Keywords:** Measurement, Arm span, Children

## Abstract

**Background:**

Arm span is an important measure for the assessment of growth and hormone deficiency diseases. In an epidemiological survey, with a large number of subjects’ indicators, it is especially valuable to establish methods which can measure both quickly and accurately. However, there are various methods, and the length of arm span may vary according to the medical institution.

**Methods:**

The arm span of nine 6-year old subjects was measured using two institutional standard methods, A and B, and a third method C which is an improved method and has been used for the first time in this study. A, No-Wall, with heels together the child stretches the arms out to the sides. B, Wall & No-Line, the child stands against the wall with heels together and spreads the arms against the wall. C, Wall & Line, the method is the same as B except a paper with horizontal lines is placed on the wall. We measured twice by each method.

**Results:**

The difference between the 1st and 2nd measurements was marginally significantly smaller by using method of C.

**Conclusion:**

The method C, which we improved, is the best way to measure arm span.

## Introduction

Arm span is an important measure in the assessment of short-limbed short stature (or long-limbed high height) resulting from growth hormone deficiency, chromosomal disorders (Turner syndrome, Marfan syndrome, etc.) and skeletal dysplasia (achondroplasia, hypochondroplasia, rickets and so on) in children [1–4]. In an epidemiological survey, with a large number of subjects’ indicators, it is especially valuable to establish methods which can measure both quickly and accurately. However, methods used to measure arm span vary among medical institutions, as do the resulting measurements.

For adults and older children, the easiest method to measure arm span is by doubling the distance between the sternal notch and the tip of the middle finger of an extended arm [5]. A second method to measure arm span, which is better for younger children, is to position the participant’s back against the wall with his/her arms spread against the wall at shoulder level and parallel to the floor with the palms facing forward. A steel measuring tape is used to measure the distance from the tip of the middle finger on one hand across the chest to the tip of the middle finger on other hand [6]. For infants, it is sometimes difficult to stretch out the fingertips and spread the arms. In such cases, they are laid on the floor and arm span is measured [7].

It is important that an accurate, consistent, and efficient method of measuring arm span width be established. Here we report on an efficient arm span measurement method that features improved accuracy.

## Methods

### Study participants

The nine children were participants in the pilot study cohort of the Japan Environment and Children’s Study, a government-funded prospective birth cohort study in Japan [8]. One hundred and five mothers and their children were included in this trial. All of the children aged 6 years. Each visited the University of Occupational and Environmental Health (UOEH) for a physical measurement as part of the follow-up program in May or June 2016.

### Arm span measurement methods

**Fig. 1:**
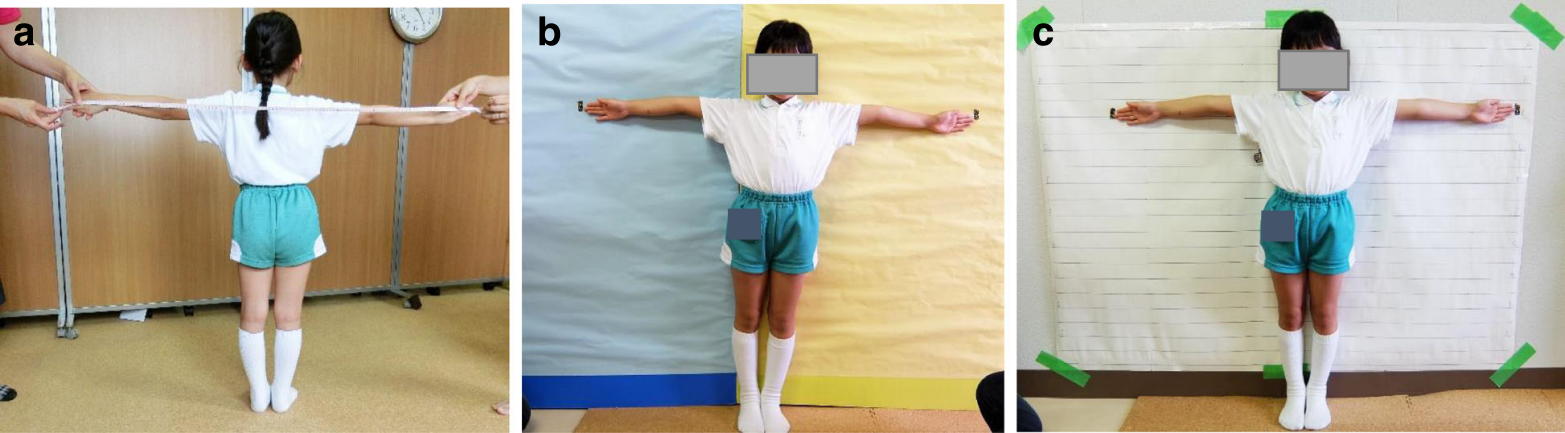
The methods of arm span measurement. **a** No-Wall. With heels together, the child stretches the arms out to the sides. **b** Wall & No-Line. The child stands against the wall with heels together and spreads the arms against the wall. **c** Wall & Line. The method is the same as B except that a paper with horizontal lines is placed on the wall

Two methods, A and B, were compared to the improved method C introduced here. In method A, No Wall, the child stood with the heels together and stretched the arms out to the sides with the palms facing forward. One investigator checked that the arms were parallel to the floor. The other two investigators measured arm span, the distance between the tips of the middle fingers across the back of the child. In method B, Wall & No Line, the child stood against a wall with the heels together and the arms spread against the wall at shoulder level parallel to the floor with the palms facing forward. One investigator checked that the arms were parallel to the floor. Two investigators placed plastic tape on the wall at the tips of the middle fingers of both hands. The investigator then measured the distance between the plastic tape marks on the wall. Method C, Wall & Line, was the same as B except a paper with horizontal lines (5-cm interval) was placed on the wall (Fig. 1).

A set of three measurements was collected twice by the same three investigators using methods C, A, and B. For each method, we also measured the time that the children must hold a constant posture: A, child holds the heels together until the measurement was over; and B and C, child keeps the heels together and against the wall for the entire measurement. Comparisons of three methods was conducted by Friedman test. Statistical analyses were performed by R 3.2.2 (http://www.r-project.org/) and all P values presented are two-sided (α = 0.05).

## Results

The median (95% confidence interval [CI]) height and weight of boys (*N =* 4) was 117.1 (108.4–124.0) cm and 21.5 (18.2–29.9) kg, respectively. The girls (*N =* 5) had a median height and weight of 109.5 (103.7–114.5) cm and 17.2 (14.3–21.3) kg, respectively.

The median (95% CI) arm spans were: No Wall (method A), first set 108.0 (105.3–115.5) cm and second set 107.5 (106.4–114.6); Wall & No Line (method B), first set 108.5 (104.3–113.9) cm and second set 107.8 (104.3–112.2); and Wall & Line (method C), first set 107.3 (103.6–112.2) cm and second set 107.5 (103.8–111.9). Table 1 compares the three measurement methods. There was a marginally significant difference among the three methods (*p =* 0.062 by Friedman test). Post-hoc test revealed a significant difference in the results between methods C and A (*p =* 0.048), but there was no difference among other comparisons.

**Table 1:** Comparison of three measurement methods

		A (No-Wall)	B (Wall & No-Line)	C (Wall & Line)	*P* value^*^
Difference between 1st and 2nd measurements (cm)	Median (95% CI)	1.0 (0.4–1.1)	0.4 (0.2–0.7)	0.2 (0.1–0.5)	0.062
The mean of the two measurement times (s)^a^	Median (95% CI)	19.1 (15.9–31.2)	19.0 (15.1–24.7)	18.0 (13.4–27.6)	0.368

*N =* 9

*CI* confidence interval

^a^The time for which a child maintains a posture

^*^Friedman test

The measurement time of method C was the smallest; however, there was no significant difference between the measurement time for each of the three methods (*p =* 0.368 by Friedman test).

## Discussion

Measurement method C was an improvement over methods A and B. Regarding measurement time, A was the longest and C was the shortest. Based on the required method preparations, we collected the measurements in the order of methods C, A, and B. The children become familiar with the actions, such putting their heels together while spreading their arms. Thus, we expected that the measurement time for C may be even shorter if it is performed later in the measurement order.

In this study, we did not use the easiest method to measure arm span, ie, doubling the distance between the sternal notch and the tip of the middle finger of an extended arm. Instead, we used method A because it was easy for the 6-year-old children to repeat spreading their arms in all three methods.

The earlier children with growth hormone deficiency, Turner syndrome, or achondroplasia/hypochondroplasia received growth hormone therapy, the higher their adult height is. Therefore, it is important to notice the sign of these diseases early by using “accurate arm span measurement”. Of course in a cohort study, the use of an accurate and efficient method for physical measurements is necessary. Method C, which used a paper with lines on the wall, was an accurate method that all medical institution can easily adopt without incurring additional cost. Changing the line spacing according to participant age and writing numbers or letters on every line in the future might make the measurements even more efficient and accurate.

## Conclusion

The method C, which used a paper with horizontal lines on the wall, is the best way to measure arm span.

## Abbreviation

UOEH: University of Occupational and Environmental Health
